# A modification to geographically weighted regression

**DOI:** 10.1186/s12942-017-0085-9

**Published:** 2017-03-31

**Authors:** Yin-Yee Leong, Jack C. Yue

**Affiliations:** Department of Statistics, National Chengchi University, Taipei, 11605 Taiwan, ROC

**Keywords:** Geographically weighted regression, Modifiable areal unit problem (MAUP), Generalized additive model, Computer simulation, Cross validation

## Abstract

**Background:**

Geographically weighted regression (GWR) is a modelling technique designed to deal with spatial non-stationarity, e.g., the mean values vary by locations. It has been widely used as a visualization tool to explore the patterns of spatial data. However, the GWR tends to produce unsmooth surfaces when the mean parameters have considerable variations, partly due to that all parameter estimates are derived from a fixed- range (bandwidth) of observations. In order to deal with the varying bandwidth problem, this paper proposes an alternative approach, namely Conditional geographically weighted regression (CGWR).

**Methods:**

The estimation of CGWR is based on an iterative procedure, analogy to the numerical optimization problem. Computer simulation, under realistic settings, is used to compare the performance between the traditional GWR, CGWR, and a local linear modification of GWR. Furthermore, this study also applies the CGWR to two empirical datasets for evaluating the model performance. The first dataset consists of disability status of Taiwan’s elderly, along with some social-economic variables and the other is Ohio’s crime dataset.

**Results:**

Under the positively correlated scenario, we found that the CGWR produces a better fit for the response surface. Both the computer simulation and empirical analysis support the proposed approach since it significantly reduces the bias and variance of data fitting. In addition, the response surface from the CGWR reviews local spatial characteristics according to the corresponded variables.

**Conclusions:**

As an explanatory tool for spatial data, producing accurate surface is essential in order to provide a first look at the data. Any distorted outcomes would likely mislead the following analysis. Since the CGWR can generate more accurate surface, it is more appropriate to use it exploring data that contain suspicious variables with varying characteristics.

**Electronic supplementary material:**

The online version of this article (doi:10.1186/s12942-017-0085-9) contains supplementary material, which is available to authorized users.

## Background

The data collected nowadays are diversified and many of them possess the records of locations, namely spatial data. Spatial regression is a popular tool for analysing the spatial data [2, 35] and the first-order stationarity is a common assumption, which means that the expected (mean) values are fixed at different locations. The error terms of spatial regression are usually not independent and, like in time series analysis, their covariance is assumed to follow some spatial models, such as the simultaneous autoregressive (SAR) and moving average (MA) models [12, 30, 34]. However, the first-order stationarity is a questionable assumption in practice and the modifiable areal unit problem (MAUP) often occurs [5, 13, 22]. The MAUP is a spatial version of the Simpson paradox, where the trends appearing in individual groups of data are different to those in the aggregate data. The biased estimates might be a consequence of the parameter values not being identical in the study area and the inclusion of data with different attributes.

Because the parameter values are not identical at different locations, estimation via the ordinary least squares (OLS) with all observations would likely distort the local distinctness. One possible solution is to include only the locations of data with similar attributes (i.e., homogeneity). However, it is difficult to decide the number of groups with different attributes and identify the locations of data in each group. Moreover, the mean value of a non-stationary process is usually a step function [8] or is continuous across space, and it is difficult to find the exact boundary of appropriate locations. The other possibility is to use the varying coefficient model [10], allowing the coefficient terms to vary according to locations. Then, the model is a form of local linear models [15] and can be used to explore the dynamic property of spatial data. Based on the concept of the varying coefficient model, geographically weighted regression (GWR) is modified to solve the MAUP [6].

The GWR allows the regression coefficients to vary across space, and the coefficient estimates of all variables are obtained from a moving data window, which is analogous to kernel regression for obtaining a smoothing estimate. It is also a popular tool for exploratory data analysis (EDA) on spatial data [19, 32]. In particular, the GWR is often a popular visualization tool in geographical information system, to explore possible patterns of a study region and acquire valuable information for further data analysis (such as clusters detection) [11, 36]. Note that the optimal width (or bandwidth) of the moving windows in a GWR is determined by cross-validation (CV) or Akaike’s information criterion (AIC) [16]. The OLS can be treated as a special case of the GWR with a window of infinite width (although the local distinctness is likely to be lost by averaging all observations).

Most of the modifications to the GWR are on the selection and testing of the bandwidth. For example, using the CV and AIC for the bandwidth selection is a data-driven method, similar to the kernel regression method, wherein the estimates are sensitive to outliers [16]. In addition, the data variations are not necessarily the same and a fixed bandwidth is likely to create discrepancy in parameters’ estimates at different locations. On the other hand, the hypothesis testing of the parameters depends on the bandwidth as well. For example, Leung et al. [21] proposed goodness-of-fit tests and found that the degree of freedom of GWR residuals is a function of the bandwidth, and this makes bandwidth selection somewhat subjective.

Determining the bandwidth probably is the focus of modifying GWR over the years. Brunsdon et al. [7] introduced a mixed GWR model with vector bandwidths, allowing the coefficients having different bandwidths (via a backfitting algorithm) and the bandwidths being functions of data density. Shi et al. [27] suggested the weight of data determined by their attributes, rather than by the distance between observations. Furthermore, Farber and Páez [16] found that it is possible to reduce the bias by modifying the CV procedure. Subsequently, Wang et al. [31] introduced local linear estimation, or a polynomial fitting technique, to reduce the bias in parameters’ estimates.

**Fig. 1:**
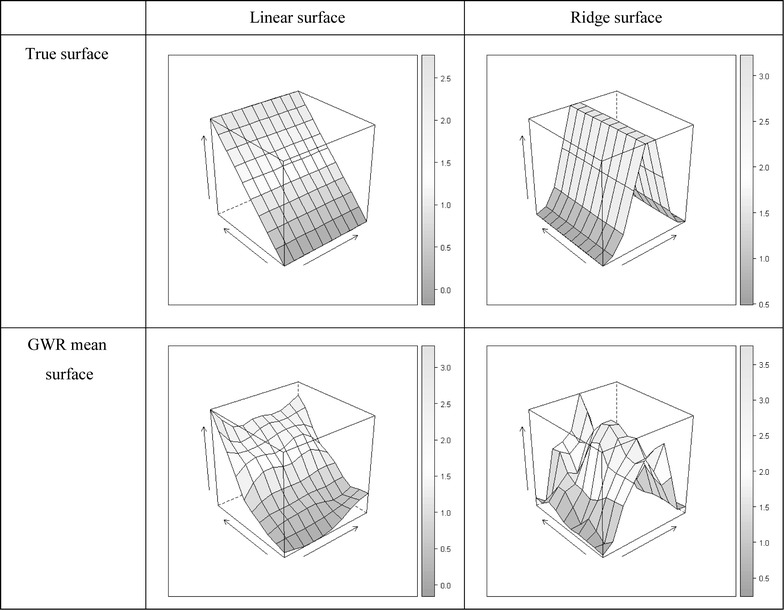
True surfaces and GWR mean surface. The figure compares the true surfaces and the estimated GWR respond surface (linear and ridge surface)

The reason for considering different bandwidth is that the GWR tends to produce ragged surfaces, in addition to biased estimates. Suppose the true surfaces are linear or ridged. As shown in Fig. 1, there are (false) hot spots and (false) cold spots in the GWR estimation, and they are especially obvious at edges and corners, where the true values and the estimated surfaces are in the first and second rows, respectively. The bias of GWR becomes larger for ridge surfaces (and other non-linear surfaces) and the GWR seems to provide misleading interpretations. The detailed discussions of GWR estimates are given later in this manuscript.

In this study, our focus is also on the bandwidth selection for each variable, using the correlations between independent variables. The idea of the proposed approach is to use the correlations to improve the estimation via an iteration algorithm, similar to the method of control variate in variance reduction [24]. The empirical analyses of GWR showed that correlations often exist between GWR coefficients. For example, Bivand and Brunstad [4] found the coefficients to be highly correlated in a case study. We found the rough coefficient surfaces can be smoother (Fig. 1) if the coefficients are positively correlated.

For the rest of this manuscript, we first introduce the GWR and the proposed modification of GWR, conditional GWR (CGWR), and its theoretical results. Then, we use simulation to evaluate the proposed method and compare it with the basic GWR and the local linear method proposed by Wang et al. [31]. In addition to simulations, we also apply the proposed method to two data sets for empirical study. Finally, we conclude with discussions on the limitations and future applicability of the proposed method.

## Methods

The GWR models a dependent variable *y* via a linear function of a set of *p* independent variables, $x_{1} ,x_{2} , \ldots ,x_{p}$, or

1 $$Y_{i} = \beta_{i0} + \sum\limits_{k = 1}^{p} {\beta_{ik} x_{ik} } + \varepsilon_{i}$$

where $\beta_{ik}$ and $x_{ik}$ are the parameters and observed values of the independent variable *k* $(k = 1, \ldots ,p)$ for observation *i*;$\varepsilon_{i}$ is the error term for observation *i*, which is generally assumed to be from a normal distribution with zero mean and constant variance $\sigma^{2}$(i.e., $\varepsilon_{i} \sim N(0,\sigma^{2} )$). The subscript *i* represents the spatial location of the observation *i* $(i = 1, \ldots ,n)$. In other words, each location has its own regression model in the GWR model. The idea behind Eq. (1) is that nearby data of each location usually possess similar attributes. Thus, choosing an appropriate range (which is referred to as “bandwidth” in this study) is plausible to obtain a fine local regression.

The parameter set $\varvec{\beta}_{\varvec{i}}$ of observation *i* is derived by matrix algebra, or

2 $$\widehat{\varvec{\beta}}_{\varvec{i}} = \left( {{\mathbf{X}}^{{\mathbf{T}}} {\mathbf{W}}_{{\mathbf{i}}} {\mathbf{X}}} \right)^{ - 1} {\mathbf{X}}^{{\mathbf{T}}} {\mathbf{W}}_{{\mathbf{i}}} {\mathbf{Y}}$$

where $\hat{\varvec{\beta }}_{\varvec{i}} = (\hat{\beta }_{i0} ,\hat{\beta }_{i1} , \ldots ,\hat{\beta }_{ip} )^{T} ,{\mathbf{X}} = ({\mathbf{1}},\varvec{x}_{1} , \ldots ,\varvec{x}_{p} )^{T} ,\varvec{Y} = ({\text{Y}}_{1} , \ldots ,{\text{Y}}_{\text{n}} )^{\text{T}}$, and ${\mathbf{W}}_{{\mathbf{i}}}$ is (diagonal) weight matrix with its weight $w_{ij}$ of row *i* and column *j* defined as:

3 $$w_{ij} = \exp \left[ { - \frac{1}{2}(d_{ij} /h)^{2} } \right]$$

As mentioned earlier, the bandwidth selection is generally calibrated by minimizing the CV score or the AIC. However, if the data locations are sparse in the study area, the distance-weighted kernel might not be appropriate due to insufficient information. Brunsdon et al. [8] introduced rank-based and k-nearest neighbourhood methods to deal with sparse data. In addition to the GWR, we also consider one of its modifications by Wang et al. [31]. It is a local linear approach, or a Taylor expansion version of the GWR, and is expected to have better fitting if the geographical surface is linear-shaped.

Using a single bandwidth in GWR is likely to create unsatisfactory estimates if the attributes of independent variables are not similar. For example, independent variables with larger variations require a larger bandwidth (and more observations). On the other hand, locally sampling (i.e. narrower bandwidth) is preferable over global sampling (i.e. wider bandwidth) for the areas with larger gradient changes. The concept is similar to widely recognized importance sampling [25], which assigns more sampling weight on informative area. Therefore, allowing different bandwidths for each independent variable seems to be a desirable modification to the GWR. Unfortunately, the varying bandwidths cannot be managed by the weighted least squares or Eq. (2). Brunsdon et al. [7] proposed a backfitting algorithm for selecting different bandwidths, but the selection of bandwidths is somewhat objective and it usually require lots of computation time.

In this study, we introduce an approach (Conditional GWR; CGWR) to determine the bandwidth for each independent variable by iteration, which is inspired by the vector bandwidth method of Brunsdon et al. [7] and the kernel smoothing method in the varying-coefficient model of Wu and Chiang [33]. For the proposed method, we adapt the ideas of the Generalized Addictive Model (GAM) and Jacobi iteration [17, 20, 23, 28] to determine the appropriate bandwidths. Using the format of the GAM, the GWR model can be re-expressed as

4 $$Y_{i} = f_{i1} + \cdots + f_{ik} + \cdots + f_{ip} + \varepsilon_{i}$$

where $f_{ik} = \beta_{ik} \times x_{ik}$ and $\beta_{ik}$ is the parameter coefficient of the variable *k* at location *i*. If $f_{ik}$ is the intercept, then $x_{ik}$ is set to 1. Then, we can use the Jacobi iteration to solve the Eqs. (4), one-by-one, for parameter $f_{ik}$. Again, we assume that $\varvec{f}_{k} \{ l\}$ denotes the *l*th iteration vector of $\varvec{f}_{k}$, and $\varvec{f}_{k} \{ l\}$ denotes an n × 1 vector composed of $f_{k}$. Then, the proposed method can be summarized iteratively as follows:

- Step 1. Set the initial solution of $\varvec{f}_{k}$ to be zero, i.e. $\varvec{f}_{k} \{ 0\} = {\mathbf{0}}$, where $k = 1, \ldots ,p$, and let $l = 1$.
- Step 2. For each element $\varvec{f}_{k} \{ l\}$, apply the basic GWR model with only one independent variable, $x_{k} .$ The value of the dependent variable is $$\varvec{y}^{*} = \varvec{y} - \sum\nolimits_{\begin{subarray}{l} j = 1 \\ j \ne k \end{subarray} }^{p} {\varvec{f}_{j} \{ \varvec{l} - {\mathbf{1}}\} }$$, i.e. we regress $$\left( {\varvec{y} - \sum\nolimits_{\begin{subarray}{l} j = 1 \\ j \ne k \end{subarray} }^{p} {\varvec{f}_{j} \{ l - 1\} } } \right)$$ on the variable $\varvec{x}_{k}$ without a fitting intercept. The bandwidth is obtained by minimizing the cross-validated sum of squares (CVSS) or the AIC.
- Step 3. Repeat Step 2 until the given stopping criterion is reached.

There are at least two reasons for finding optimal bandwidth solutions individually using the Jacobi iteration. First, although more complex numerical methods (such as the quasi-Newton method) could be used, the Jacobi iteration usually requires less computation time. Second, although there are algorithms that converge faster than the Jacobi iteration, they are likely to produce biased estimates. For example, in the Gauss–Seidel iteration process, the estimate of one variable is updated based on the simultaneous estimates of other variables. If the estimates of some variables have severe biases, it might contaminate the estimates of other variables.

We think that the proposed estimation process can guarantee the convergence of the CGWR. In particular, if the bandwidth is predetermined during the iteration, then the GWR coefficients will converge to a constant for each location. We should use the case of two coefficients to demonstrate the convergence and the outline proof is given in Appendix A in Additional File 1. Note that the method of Brunsdon et al. [7] can be treated as a special case of the CGWR method when the bandwidths are never updated. In the next section, we will use computer simulation to evaluate the stability of the CGWR and compare it with basic GWR and its local linear modification by Wang et al. [31].

## Results and discussion

### Simulated data

The computer simulation is separated into two parts: scenarios without clusters and with a cluster. For the latter scenario, a cluster is added into the intercept to exhibit the mean shift intervention. The cluster scenario is to evaluate the performance of estimation methods under the influence of a systematic change (or hot spots) in space, such as sources of pollution. Moreover, the coefficients are assumed to follow one of the following four surfaces: linear, quadratic, ridge, or hillside, and these settings are to check which would cause raggedness in the estimated surfaces. For the former scenario, we also examine two types of surfaces: single-type and mixed-type. The difference between these two types of surfaces is whether the coefficients follow same type of surfaces (single-type) or different types of surfaces (mixed-type). We want to know if the coefficients follow different types of surface would cause biased estimation of coefficients.

To simplify the discussion, suppose there are only two coefficients, i.e. one intercept and one independent variable in the spatial regression, or

5 $$Y_{i} = \beta_{i0} + \beta_{i1} x_{i} + \varepsilon_{i} ,$$

where *i* is a natural number which indicates the location of the observation. Next, we define the signal versus noise ratio, i.e. the S/N ratio, where the signal represents the variations on the surface of the coefficients and the noise is the random fluctuation of observations. Larger S/N ratios are associated with larger variations in the coefficient surfaces, in which case the coefficient’s pattern is easier to be detected. In particular, we assume that signal = $3 \times \left( {\frac{{\sum\nolimits_{i} {(\beta_{ik} - \bar{\beta }_{k} )^{2} } }}{n - 1}} \right)^{1/2}$ and the noise is equal to the standard deviation of the error term. Here, it is 0.5. The bias and variance of the estimates can be used together to evaluate the accuracy of the proposed CGWR. We define the average discount rate as follows:

6 $$\frac{{n^{ - 1} \sum\nolimits_{i} {(MSE_{method} )} }}{{n^{ - 1} \sum\nolimits_{i} {(MSE_{OLS} )} }},$$

where MSE refers to the mean square error, i.e. the sum of variance and the squared bias. Note that the MSE of the OLS estimate is used as a benchmark for comparison in Eq. (6) and it can be computed for all locations by using the weighted mean given by:

7 $$\frac{{\sum\nolimits_{i} {\left[ {(MSE_{OLS} )_{location \, i} \times \left( {MSE_{method} /MSE_{OLS} } \right)_{location \, i} } \right]} }}{{\sum\nolimits_{i} {(MSE_{OLS} )_{location \, i} } }} .$$

**Fig. 2:**
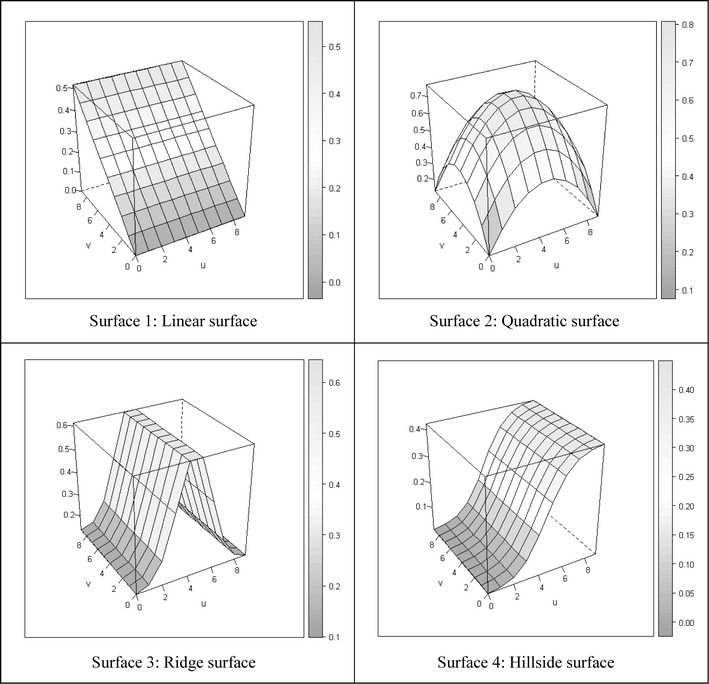
Four coefficient surfaces. The figure illustrates different kinds of surfaces in simulation settings (1 and 2 are Polynomial, and 3 and 4 are non-polynomial)

There are four types of surfaces for the coefficients, as shown in Fig. 2. Surfaces 1 and 2 are polynomial functions (related to linear functions) of the independent variables, and surfaces 3 and 4 are non-linear. Similar settings also appear in previous studies on GWR [30], and these have practical implications. For example, the quadratic surface (surface 2) often occurs in situations involving housing prices, where prices are significantly higher for locations near a town centre or a transportation centre [14, 31]. In addition, the relationship between the environmental factor and real estate price can be different in urban areas than in the countryside [9]. Furthermore, the relationship between disease and environmental factors might appear as non-linear on geographic surface. For instance, incidence rate of Dengue disease are highly correlated with population density but the relationship seems to fade out if there is proper disease prevention policy [26]. As a result, the surface of coefficients can be non-linear. For every non-stationary surface, we assume that there are 10 × 10 regular lattice points (i.e. 100 locations).

For a scenario without clusters, several cases are tested under different S/N ratios. The first case is the single-type surface, where both intercept and independent variable follow the same type of surface. The second case, namely mixed-type surface, assumes that the intercept and the independent variable follow different types of surfaces. For the second case, we only examine two combinations: linear-quadratic (a polynomial surface) and ridge-hillside (a non-polynomial surface).

For the scenario with clusters, we add two clusters in an intercept. The clusters are circular and occupy 18% of space in the study area. The circular assumption is quite usual in geographical studies, and literature proves that 10–20% of clustered area is a common phenomenon [29]. Two levels of mean shifts, such as 1σ and 2σ,are also added at the cluster locations. The simulation settings of both scenarios are in Table 1. For all scenarios, the errors are drawn from a normal distribution with a mean of 0 and a standard deviation of 0.5. The reason for choosing the standard deviation 0.5 is to incorporate the values of the S/N ratio. All results are based on 100 simulation runs.

**Table 1:** The scenario settings of simulation study

	Mean shift intervention	
	No cluster	Cluster
*Surfaces type*		
I. Single-type surface	1. Linear, 2. quadratic, 3. ridge, 4. hillside	1. Linear, 2. hillside
II. Mixed-type surface	1. (Linear, quadratic) 2. (ridge, hillside)	–
*Fitting method*		
Two-stage fitting procedure	1. Linear, 2. hillside	–

There are totally 6, 4, and 2 scenarios in the Single-type surface, Mixed-type surface, and Two-Stage Fitting procedure, respectively. The bracket in the Mixed-type surface scenario indicates the surfaces of intercept and variable *x*_1_ separately

For CGWR, the Gaussian kernel is chosen, and the optimal bandwidth is the one with the minimum CVSS. Furthermore, we require a reasonable range of bandwidth to prevent the estimation from being too localized or globalized for extremely small or large bandwidths, respectively. The upper bound of the range is the maximum length on the map, and the lower bound has to have at least five data points each of 1/5th weight. The preceding setting is also used in the ‘spgwr’ package [3] (version 0.5–4) of R, a free statistical software.

We will first show the simulation results of the scenario without clusters. In particular, we will compare three GWR estimations with the smoothness of the mean surface, the average discount rate, average bandwidth, average variance, and average bias of estimates. The stopping criterion for the CGWR is reached when the average absolute relative change rate of $\beta_{0}$ and $\beta_{1}$ is less than 0.005% of the previous step. The simulation results are similar if adopting smaller stopping criteria.

To simplify the notation, we will use $\beta_{0}$ and $\beta_{1}$ to denote the coefficients of the intercept and slope of the independent variable *x*, respectively. In the case of single-type surfaces and mixed-type surfaces, the two coefficients are assumed to be perfectly positively correlated and close to uncorrelated, respectively. We have not considered the negative correlation because the proposed algorithm does not work when the coefficients are not positively correlated. Nonetheless, we will consider a two-stage modification for CGWR when the coefficients are not positively correlated.

#### Single-type surfaces

**Fig. 3:**
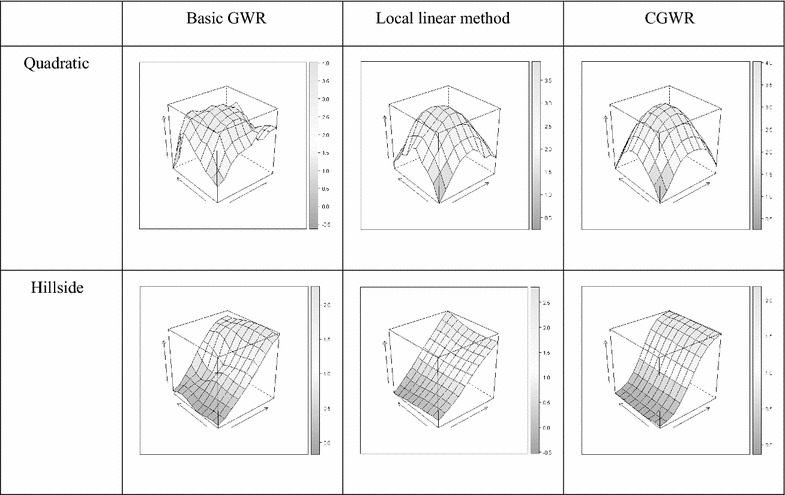
$\beta_{ 0}$ Mean surface. Estimated intercept respond surface derived from different method (quadratic and hillside surfaces, S/N ratio of $\beta_{ 0}$ = $\beta_{1}$ = 5)

**Fig. 4:**
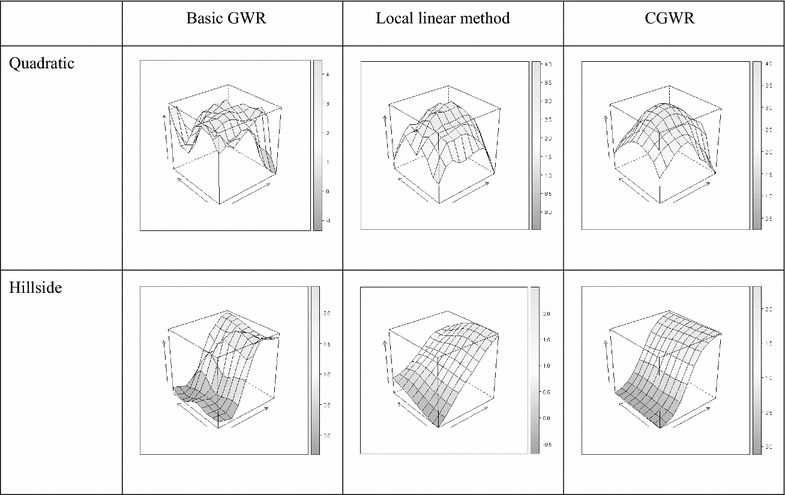
$\beta_{ 1}$ Mean surface. Estimated intercept respond surface derived from different method (quadratic and hillside surfaces, S/N ratio of $\beta_{ 0}$ = $\beta_{1}$ = 5)

We first compare the smoothness of the three different GWR methods. For instance, the mean surfaces from 100 simulation runs for quadratic and hillside surfaces with the S/N ratio of $\beta_{0}$ = $\beta_{1}$ = 5 are shown in Figs. 3 and 4. Obviously, the CGWR produces the best fit, and the mean surfaces are almost identical to the true surfaces as shown in Figs. 2, 3, and 4. The GWR tends to produce bumpy surfaces, which are more ragged for the $\beta_{1}$ surfaces. The edge effect of the GWR is obvious; this may be because fewer observations were used in the estimation. On the other hand, the local linear method tends to produce linear-like surfaces and provides distorted information for the non-linear surfaces and for the $\beta_{1}$ surfaces. In contrast, the CGWR produces a remarkable fit even in complex surfaces and provides valuable information for further data analysis.

Table 2 shows the results of the discount rates in the case where $\beta_{0}$ and $\beta_{1}$ follow a linear surface. We can see that both the proposed CGWR and the local linear method have significant improvements over the basic GWR method. Interestingly, the local linear method is better (with respect to smaller discount rates) than the GWR when the S/N ratio is large, but the basic GWR is better when the S/N is small. The reason might be that larger noises produce larger fluctuations, and thus the average tangent line in the local linear method is inaccurate or unstable. Similar results are also found for the other three surfaces, as evidenced in Tables 3, 4, and 5. This suggests that the local linear method might not be very stable if the S/N ratio is small.

**Table 2:** Average discount rates on a linear surface (single-type)

S/N ratio		$\beta_{ 0}$			$\beta_{1}$		
	$\beta_{ 0}$	1	3	5	1	3	5
	$$\beta_{1}$$						
Basic GWR	1	1.064	0.312	0.158	1.815	1.589	1.328
	3	1.348	0.321	0.173	0.598	0.663	0.755
	5	1.775	0.382	0.199	0.329	0.397	0.459
Local linear	1	1.317	0.192	0.078	2.111	1.033	0.686
	3	1.107	0.164	0.069	0.403	0.347	0.284
	5	1.299	0.188	0.059	0.197	0.179	0.126
CGWR	1	0.664	0.195	0.082	1.076	0.775	0.412
	3	0.880	0.202	0.086	0.400	0.366	0.286
	5	0.899	0.227	0.092	0.198	0.207	0.178

The values (1, 3, 5) indicate the signal/noise ratio

**Table 3:** Average discount rates on a quadratic surface (single-type)

S/N ratio		$\beta_{ 0}$			$\beta_{1}$		
	$\beta_{ 0}$	1	3	5	1	3	5
	$$\beta_{1}$$						
Basic GWR	1	1.955	0.791	0.510	2.877	7.729	7.542
	3	3.217	0.896	0.570	1.418	2.332	3.650
	5	4.334	1.108	0.639	0.844	1.316	1.819
Local linear	1	3.279	0.914	0.535	5.159	8.900	7.941
	3	5.177	1.119	0.599	2.039	2.634	3.481
	5	6.147	1.196	0.602	1.001	1.169	1.488
CGWR	1	1.168	0.324	0.146	1.529	1.612	0.928
	3	1.491	0.380	0.166	0.675	0.713	0.730
	5	1.709	0.429	0.183	0.374	0.451	0.430

The values (1, 3, 5) indicate the signal/noise ratio

**Table 4:** Average discount rates on a ridge surface (single-type)

S/N ratio		$\beta_{ 0}$			$\beta_{1}$		
	$\beta_{ 0}$	1	3	5	1	3	5
	$$\beta_{1}$$						
Basic GWR	1	1.740	0.571	0.383	2.642	2.325	2.249
	3	2.623	0.728	0.401	1.150	1.375	1.321
	5	2.892	0.824	0.433	0.591	0.810	0.894
Local linear	1	2.890	1.023	0.621	4.745	4.374	3.672
	3	4.340	1.206	0.618	1.960	2.255	2.023
	5	5.385	1.386	0.677	1.081	1.326	1.344
CGWR	1	0.884	0.203	0.111	1.029	0.428	0.258
	3	1.048	0.239	0.116	0.529	0.385	0.250
	5	0.854	0.221	0.107	0.253	0.208	0.159

The values (1, 3, 5) indicate the Signal/Noise Ratio

**Table 5:** Average discount rates on a hillside surface (single-type)

Signal/noise ratio		$\beta_{ 0}$			$\beta_{1}$		
	$\beta_{ 0}$	1	3	5	1	3	5
	$$\beta_{1}$$						
Basic GWR	1	1.075	0.315	0.153	1.697	1.905	1.359
	3	1.353	0.340	0.173	0.583	0.603	0.668
	5	1.152	0.343	0.185	0.234	0.278	0.360
Local linear	1	1.339	0.243	0.149	2.187	1.549	1.239
	3	1.403	0.289	0.188	0.617	0.529	0.722
	5	1.113	0.339	0.209	0.266	0.307	0.421
CGWR	1	0.726	0.170	0.077	1.098	0.761	0.473
	3	0.926	0.199	0.079	0.384	0.348	0.239
	5	0.578	0.192	0.079	0.159	0.158	0.140

The values (1, 3, 5) indicate the signal/noise ratio

The CGWR and the local linear method again outperform the basic GWR method in the case of a quadratic surface. However, the CGWR appears to be the best, and the advantage is more obvious when the S/N is increased. For the non-linear surfaces, the CGWR continues to work satisfactorily, whereas the local linear model does not. In fact, the local linear model might even produce worse results than the basic GWR. The CGWR is still reliable for the non-linear surfaces, and it performs much better than the other two methods.

Intuitively, we expect that the bandwidth to be small if the S/N is large because distant observations can be very different and cause biased estimations. In general, all three GWR methods have significant drops in bandwidth when the S/N ratio increases from one to three. Moreover, the bandwidths for a linear surface should be larger than those for a non-linear surface under the same S/N ratio because the surface change is quite homogenous in any direction.

The bandwidth results can also be used to explain why the CGWR outperforms the other two methods. We will choose two surfaces (linear and hillside) to discuss these results. Table 6 shows the average bandwidths. The local linear method often yields larger bandwidths. If the true surface is close to linear, we can rely on observations within a larger bandwidth and, thus, have smaller variances than those for non-linear surfaces. Since the shape of a hillside is close to linear, the bandwidths in the case of the hillside are very similar to those in the linear case. They are also much larger than those of the quadratic and ridge cases. For more details, see Appendix B in Additional File 1.

**Table 6:** The average bandwidths for linear and hillside surfaces (single-type)

S/N		Linear surface			Hillside surface		
	Method	Basic GWR	Local linear	CGWR	Basic GWR	Local linear	CGWR
1		2.35	9.73	4.16; 7.24	2.25	9.78	4.72; 7.45
3		1.46	10.8	1.65; 2.31	1.47	9.01	1.57; 2.80
5		1.2	10.34	1.25; 1.47	1.24	6.04	1.25; 1.66

The value (1, 3, 5) indicate the signal/noise ratio. For CGWR, the first and second values are the average bandwidths of $\beta_{ 0}$ and $\beta_{1}$, respectively

The bandwidths of the CGWR seem to be related to the signal strength. For example, if the S/N ratio is small, the bandwidth is expected to be large in order to provide a stable estimate. If we fix the S/N ratio of $\beta_{1}$, then the $\beta_{0}$ bandwidth of the CGWR decreases as the S/N ratio of $\beta_{0}$ increases for all four surfaces (Appendix B in Additional file 1). Similar results hold for the $\beta_{1}$ bandwidths if we fix the S/N ratio of $\beta_{0}$. The simulation results of CGWR match our expectations.

The variances and biases of the estimates from the three GWR methods can also be used for making comparisons. Again, we will use the cases of linear and ridge surfaces for a detailed discussion. Further, because there are many combinations for the S/N ratios of $\beta_{0}$ and $\beta_{1}$, we have only shown the results when the S/N ratio equals one and five. The results are shown in Tables 7 and 8. Unlike the previous comparisons, we also provide the variances and biases of the OLS estimates. In general, a larger S/N ratio tends to produce a larger bias. Moreover, the OLS estimates fail to capture the spatial trend causing the largest bias, but it uses all the observations in the estimation (i.e. infinite bandwidth) and thus has the smallest variance. As for the three GWR estimations, the variances of the estimators are generally larger than the biases.

**Table 7:** Average variances and biases of $\beta_{ 0}$ and $\beta_{1}$ on a linear surface (single-type)

S/N of $\beta_{ 0}$:	OLS		Basic GWR		Local linear		CGWR	
	1	5	1	5	1	5	1	5
*(i)* $\beta_{ 0}$								
Conditional on S/N of $\beta_{1}$ = 1								
Variance	0.009	0.011	0.039	0.067	0.051	0.052	0.024	0.036
Bias	0.029	0.028	0.002	0.004	0.0003	0.0002	0.001	0.0002
Conditional on S/N of $\beta_{1}$ = 5								
Variance	0.010	0.009	0.090	0.107	0.057	0.043	0.048	0.057
Bias	0.723	0.719	0.026	0.038	0.0005	0.0002	0.012	0.009
*(ii)* $\beta_{1}$								
Conditional on S/N of $\beta_{1}$ = 1								
Variance	0.031	0.036	0.113	0.190	0.146	0.145	0.071	0.117
Bias	0.038	0.695	0.013	0.051	0.001	0.0005	0.004	0.028
Conditional on S/N of $\beta_{1}$ = 5								
Variance	0.030	0.027	0.248	0.294	0.152	0.114	0.088	0.152
Bias	0.191	0.880	0.048	0.124	0.001	0.0002	0.004	0.010

The values (1, 5) indicate the signal/noise ratio

**Table 8:** Average variances and biases of $\beta_{ 0}$ and $\beta_{1}$ on a hill-side surface (single-type)

S/N of $\beta_{ 0}$	OLS		Basic GWR		Local linear		CGWR	
	1	5	1	5	1	5	1	5
*(i)* $\beta_{ 0}$								
Conditional on S/N of $\beta_{1}$ = 1								
Variance	0.008	0.008	0.031	0.060	0.044	0.053	0.026	0.040
Bias	0.028	0.702	0.003	0.033	0.002	0.033	0.002	0.009
Conditional on S/N of $\beta_{1}$ = 5								
Variance	0.009	0.008	0.053	0.080	0.048	0.061	0.025	0.046
Bias	0.027	0.703	0.003	0.042	0.003	0.041	0.001	0.008
*(ii)* $\beta_{1}$								
Variance	0.023	0.021	0.090	0.183	0.115	0.178	0.054	0.084
Bias	0.034	0.129	0.013	0.107	0.007	0.031	0.004	0.009
Conditional on S/N of $\beta_{1}$ = 5								
Variance	0.024	0.022	0.168	0.239	0.159	0.192	0.110	0.106
Bias	0.068	0.811	0.049	0.179	0.045	0.097	0.013	0.033

The values (1, 5) indicate the signal/noise ratio

The results of the linear surface are in Table 7. As mentioned earlier, the average bandwidths of the local linear method are the largest, which possibly indicates the smallest variances. In addition, the local linear method has the smallest bias and the smallest discount rates for linear surfaces (Table 2). Although the CGWR has a larger bias than the local linear method in the case of linear surface, it dominates the basic GWR method with respect to both the variance and bias. The CGWR performs the best with ridge surfaces, outperforming the basic GWR and the local linear method with respect to both variance and bias.

#### Mixed-type surfaces

Next, we repeat the same comparisons for the three GWR estimation methods with the mixed-type surfaces. The results are similar to those in the single-type surfaces, and thus we will only show the results of the discount rates. As mentioned earlier, there are two cases in this scenario: linear-quadratic (a polynomial surface) and ridge-hillside (a non-polynomial surface). In the first case, the underlying surface of intercept is linear, and the slope is quadratic. In the second case, all surfaces are of non-polynomial type, and it is more complex than the first one.

Basically, the CGWR also has smaller discount rates than the basic GWR for the mixed-type surfaces (Tables 9, 10). We will focus on the results that differ from those of the single-type surfaces. Although the local linear estimation is better than the GWR for linear-quadratic surfaces, it performs adversely for the ridge-hillside surfaces. It is inadequate to use the linear fitting method to approximate non-linear surfaces, such as the ridge-hillside case. On the contrary (similar to the single-type cases) the CGWR dominates the other two methods in both cases.

**Table 9:** The average discount rates of a linear-quadratic surface (mixed-type)

		$\beta_{ 0}$			$\beta_{1}$		
	$\beta_{ 0}$	1	3	5	1	3	5
	$$\beta_{1}$$						
Basic GWR	1	1.062	0.279	0.155	1.934	1.643	1.420
	3	2.110	0.407	0.181	1.048	1.067	0.935
	5	3.119	0.537	0.231	0.688	0.687	0.711
Local linear	1	1.492	0.225	0.080	2.888	1.514	0.905
	3	3.100	0.534	0.169	1.405	1.314	0.954
	5	5.357	0.775	0.268	0.928	0.871	0.753
CGWR	1	0.764	0.183	0.100	1.301	0.802	0.538
	3	1.132	0.345	0.179	0.708	0.849	0.785
	5	1.257	0.388	0.217	0.404	0.523	0.584

The values (1, 3, 5) indicate the signal/noise ratio

**Table 10:** The average discount rates of a ridge-hillside surface (mixed-type)

		$\beta_{ 0}$			$\beta_{1}$		
	$\beta_{ 0}$	1	3	5	1	3	5
	$$\beta_{1}$$						
Basic GWR	1	1.457	0.545	0.337	2.160	3.268	2.868
	3	1.623	0.582	0.349	0.622	1.373	1.786
	5	1.511	0.604	0.344	0.251	0.586	0.943
Local linear	1	2.105	0.853	0.536	3.312	5.602	4.755
	3	2.236	0.903	0.497	0.896	2.348	2.595
	5	1.999	0.980	0.540	0.333	1.076	1.522
CGWR	1	0.956	0.245	0.116	1.166	0.750	0.351
	3	1.279	0.361	0.151	0.544	0.677	0.443
	5	1.338	0.485	0.204	0.245	0.434	0.415

The values (1, 3, 5) indicate the signal/noise ratio

#### Two-stage fitting procedure

We found that the CGWR works well when there is a positive correlation. However, in reality, there is a great likelihood of variables not being positively correlated. To overcome this difficulty, the CGWR can be modified into a two-stage process. In the first stage, we divide the variables into two groups. In both groups, the variables are non-negatively (or positively) correlated within the group. Any two variables are non-positively (or negatively) correlated if they are from different groups. We choose one group of variables and apply the basic GWR method to this group. In the second stage, we apply the CGWR method to the other group of variables by treating the first group of variables (chosen in the first stage) as constants.

**Fig. 5:**
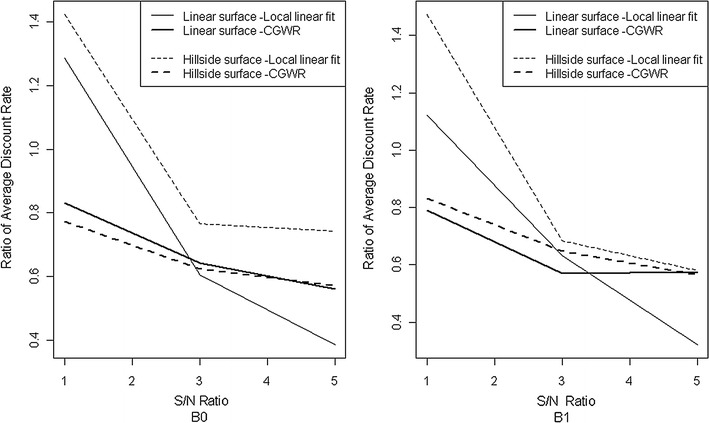
Average discount rates by $x_{2}$. The average discount rates of additional explanatory variables during the simulation study. The baseline is GWR. The ratio of average discount rate under different Signal to noise ratio. *Left panel* indicate the performance on $\beta_{0}$, and indicate performance on $\beta_{ 1}$ for *right panel*

We use an example to demonstrate the two-stage fitting. Let us assume there are two independent variables and an intercept. Let the coefficients of the two independent variables be negatively correlated. In other words, let the coefficients of variables *x*_1_ and *x*_2_ be negatively correlated, and the coefficients between *x*_1_ and intercepts be positively correlated. We first apply the basic GWR on *x*_2_ in the first stage, and then apply the CGWR on the intercept and *x*_1_ in the second stage. We will use a simulation to evaluate the two-stage modification and show the results of the linear and hillside surfaces in Fig. 5. Similar to the previous simulation, the two-stage CGWR seems to work well even when the variables are not all positively correlated.

#### Single-Type Surfaces with Clusters

**Fig. 6:**
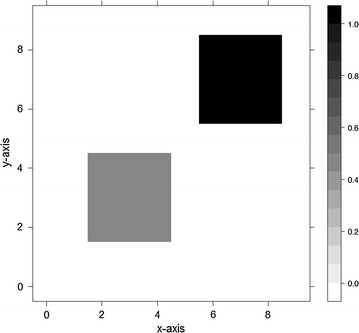
Cluster location and mean shift level. The *area* shows cluster intervention in simulation study. The *shade depth* in the *first graph* represents the mean shift level. The simulated region falls within Cartesian product within 0 and 1 (i.e. [0, 1] × [0,1]). There are two artificial clusters (hot-spots) located on lower-left and top-right. The *lower-left* cluster has lower relative risk and the *top-right* cluster has higher relative risk

**Fig. 7:**
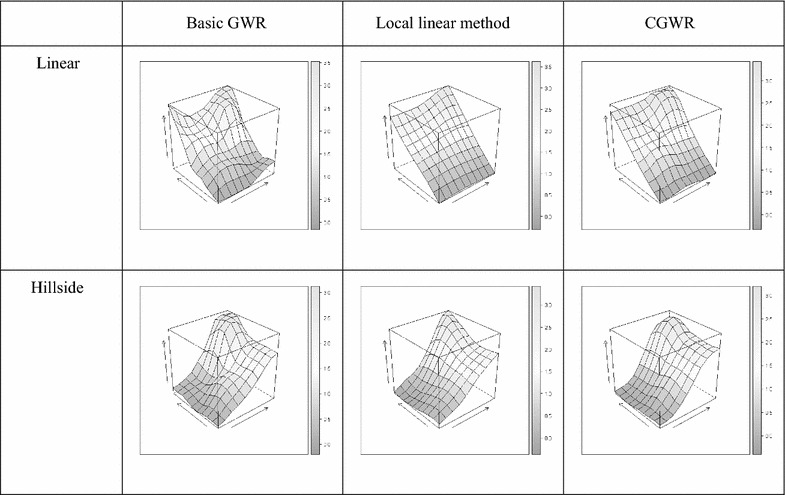
$\beta_{ 0}$ Mean surface in cluster settings. Estimated intercept respond surface derived from different method (quadratic and hillside surfaces, S/N ratio of $\beta_{ 0}$ = $\beta_{1}$ = 5)

The purpose of considering the scenario with clusters is to investigate whether the estimated surface would be influenced by the cluster intervention on $\beta_{ 0}$. Figure 6 illustrates the cluster location and the mean shift level. Single-type surfaces are assumed to evaluate the performance under cluster intervention. The mean smoothness and average discount rate of $\beta_{ 0}$ are in Fig. 7 and Table 11. The CGWR again has the best performance and provides the most accurate information pertaining to the location and size of the clusters. Although the GWR seems to reveal the true cluster locations, it suffers from bumpy fitting and gives rise to ‘false clusters.’ The local linear method seems to oversmooth the surface and blur the local pattern, although this might suggest a possible cluster on the edges.

**Table 11:** The average discount rates of $\beta_{ 0}$ on surfaces with cluster

		Linear			Hillside		
	$\beta_{ 0}$	1	3	5	1	3	5
	$$\beta_{1}$$						
Basic GWR	1	0.975	0.395	0.214	0.929	0.386	0.198
	3	1.116	0.421	0.240	0.964	0.416	0.196
	5	1.202	0.473	0.257	1.007	0.412	0.197
Local linear	1	1.166	0.402	0.207	1.142	0.543	0.284
	3	1.246	0.45	0.210	1.238	0.593	0.325
	5	1.287	0.419	0.225	1.358	0.619	0.288
CGWR	1	0.714	0.226	0.098	0.625	0.216	0.106
	3	0.943	0.302	0.133	0.852	0.281	0.112
	5	1.146	0.368	0.150	1.000	0.303	0.123

The values (1, 3, 5) indicate the signal/noise ratio

From the preceding computer simulations studies, we found that the proposed CGWR method makes a significant improvement over the basic GWR method. Although the local-linear method behaves well in the linear surface, if the coefficient surfaces are non-linear, the CGWR also outperforms the local linear method. In the following discussion, we will use two real-world datasets to compare the CGWR and other two methods and provide further evidence in support of the CGWR.

### Empirical data

We apply the CGWR to two empirical data sets: the first is from the 2000 Taiwan Census and the other is the Ohio crime data provided by Anselin [1]. These two examples are designed to demonstrate that the CGWR would yield better estimation results. For the Taiwan data, our goal is to explore the relationship between the proportion of elderly disability and social factors. The elderly population in Taiwan have been increasing rapidly all around the country, while the medical resources still concentrate in the metropolitan areas (or northern Taiwan). Hu and Yue [18] applied spatial regression model to the elderly disability data of township level and found they are spatially auto-correlated. Brunsdon [7] argued that the spatial autocorrelation seems to be caused by spatial non-stationarity (i.e., identifiability). His claim motivates us to re-examine the data using the GWR-based model.

#### Taiwan data

Taiwan 2000 Census includes data of 350 townships and their proportions of disabled elderly are set as the dependent variable. Since this variable appears to be right-skewed, a log transformation (i.e.$y_{i}^{*} = \log (y_{i} + 1)$) is applied. Four independent variables are selected: the population density (POP), proportion of elderly (ELD), elderly mortality rate (EMR), and education level (EDU). These independent variables are standardized into the [0, 1] interval. Before applying the GWR, we first test spatial non-stationarity with the *F* test suggested by Leung et al. [21]. The *F* test shows that the model is spatially non-stationary with *p* value <0.001. This confirms the hypothesis of Brunsdon [7] and creates incentive for plugging the GWR-type analysis.

The correlation of the intercept and the variable POP is 0.463 (Table 12), and they are placed in the same group. Similarly, the variables ELD, EMR, and EDU are in the other group since they are positively correlated pairwise. Thus, we use the two-stage modification and apply the CGWR on the group of positively correlated variables (i.e. the intercept and the variable POP). First, we treat the variables ELD, EMR, and EDU as constants after obtaining estimates from the basic GWR method. Then, we apply the CGWR to the intercept and the variable POP as $\left( {y_{i}^{*} - \hat{\beta }_{i2}^{GWR} ELD_{i} - \hat{\beta }_{i3}^{GWR} HMR_{i} - \hat{\beta }_{i4}^{GWR} EDU_{i} } \right) = \hat{\beta }_{i0}^{CGWR} + \hat{\beta }_{i1}^{CGWR} POP_{i} + r_{i}$. After fitting the CGWR, the calibrated bandwidths vary across the variables. Also, we set the lower and upper bounds of the bandwidth as 1 and 400 km, respectively.

**Table 12:** Correlations of regression coefficients from the disability data (INT and POP) → group 1, (ELD, EMR, EDU) → group 2

	INT	POP	ELD	EMR	EDU
INT	1.000	0.463	−0.949	−0.951	−0.971
POP	0.463	1.000	−0.281	−0.281	−0.653
ELD	−0.949	−0.281	1.000	0.914	0.875
EMR	−0.951	−0.281	0.914	1.000	0.868
EDU	−0.971	−0.653	0.875	0.868	1.000

INT represents the intercept in GWR. Other variables include Population Density (POP), Proportion of Elderly (ELD), Elderly Mortality Rate (EMR), Education (EDU)

**Fig. 8:**
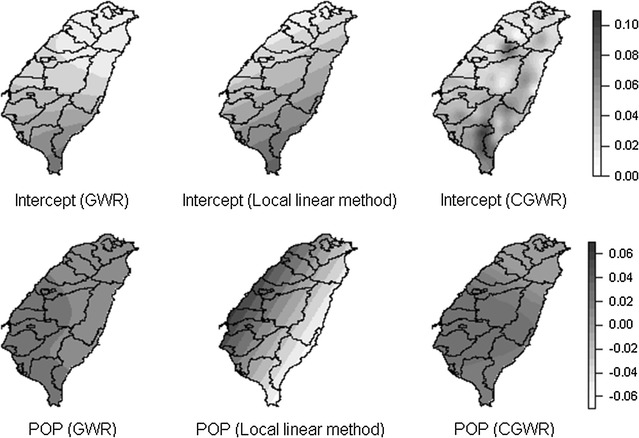
Surface of intercept and variable POP for different GWR methods. Comparison of different method by using Taiwan disability data

There is a noticeable difference between the estimates from the CGWR and those from other methods (Fig. 8). The coefficient surfaces of the local linear method appear to spread in the north–south or east–west direction with linear boundaries. Similarly, the surfaces of the basic GWR also show descending (or ascending) patterns but with curved boundaries. However, the intercept of the CGWR shows clusters (or concentration) of high disability rate on inland (mountainous areas). For the variable POP, the number of coefficient levels varies among different models and their spreading directions are not identical. The GWR has the fewest levels and the local linear method has the largest; the spreading is in the east–west direction for the local linear method, different to other methods.

**Fig. 9:**
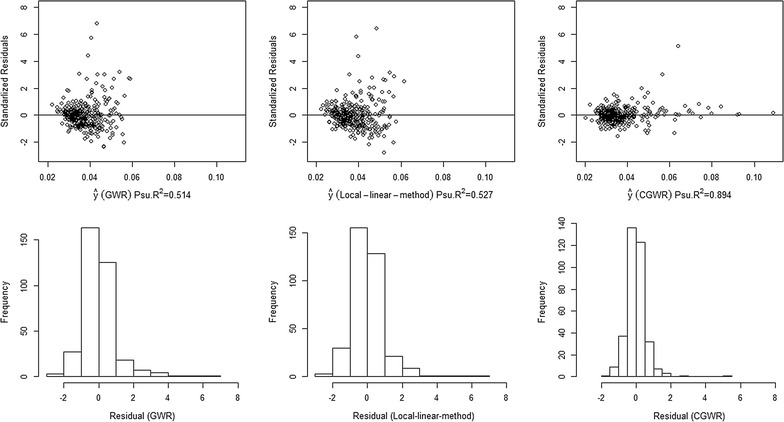
Residual plots for different GWR methods (Taiwan data). These plots illustrate the residual analysis after fitting with different model. The residuals are generated from Taiwan disability data

We also use the pseudo R-square values and the residual plots for model evaluation (Fig. 9). The pseudo R-square is the Pearson product moment correlation coefficient of the fitted value and the observed value; a large value usually indicates a better fit. The pseudo R-square value of the CGWR is 0.894, largest among three methods. Moreover, the residual plots are also in favour of the CGWR because there are fewer outliers, and the CGWR appears to have smaller variance. Except for one observation (standardized residual larger than 3), the residuals histogram of CGWR (350 observations) looks more symmetric and less skewed to the right than those of the basic GWR and local linear method. It should be noted that either one of the variable sets can be chosen as a constant. If we apply the CGWR procedure to the other group of variables (i.e. ELD, EMR, and EDU), then the CGWR has a better fit (although the pseudo R-square is slightly low at 0.874).

#### Ohio Data

The Ohio data is the Ohio crime data (which can be found on ‘spgwr’ package) with the information of 49 neighborhoods including the crime per inhabitant, average income values, and average housing costs. In this study, we define the crime per inhabitant as the dependent variable and the rest of them as the predictors. First, we fit the data with the GWR model. By cross validation criterion, the optimal bandwidth is 2.27 (Table 13). And yet, Leung et al. *F* test [21] suggests none of the variable is non-stationary. Therefore, the OLS analysis is applied and one observation is considered as outlier and removed accordingly.

**Table 13:** The bandwidths of different models

Method	Variable		
	Intercept	*x*1 (average income value)	2 (average housing cost)
Basic GWR	2.27	2.27	2.27
Local linear	13.81	13.81	13.81
CGWR	999	999	1.77

**Fig. 10:**
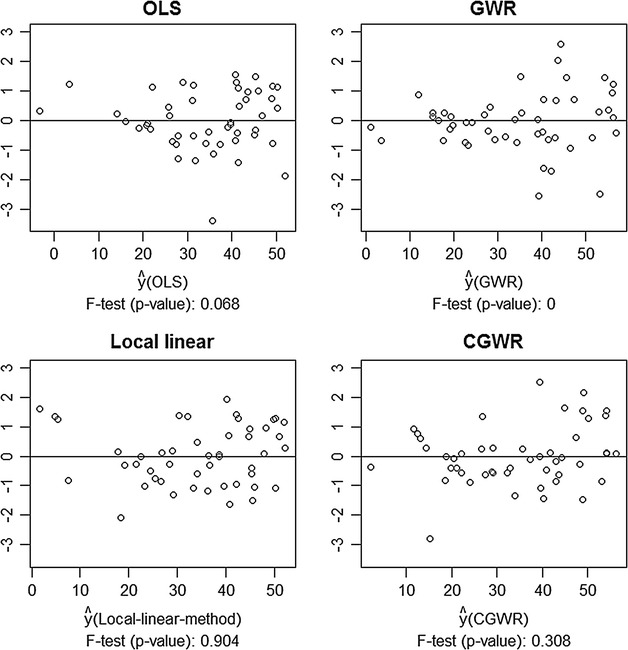
Residual plots of various models (Ohio data). Different residual plot from Ohio crime data. Note that the data are cut into half according to fitted values before conducting *F* test. The data are cut into half according fitted values before conducting constant variance test. For example, the middle point of GWR fitted values is around 29.1. We first split the data to two sets (before 29.1 and after 29.1). After that, *F* test is conducted to test whether both parts have equal variance (i.e. ${\text{H}}0:\upsigma^{1} =\upsigma^{2}$), which is the basic assumption in regression analysis. Small *p* value indicates constant variance assumption is not likely to be true

Next, we re-fit the data with the CGWR and compare the estimation result with those of the OLS, the GWR, and the local linear models. Table 14 lists the pseudo R-square and the *p* value of normality test (Kolmogorov–Smirnov test) for residuals, and Fig. 10 shows the residuals plot. Overall, the CGWR yields the best performance in estimation and produces a more reliable result. For other methods, none of them gives satisfactory estimates. For example, despite the GWR produces a large pseudo R-square, its residuals are not normally distributed and its variance is likely not constant. The OLS is also not a feasible model, judging from the information of normality test and constant variance.

**Table 14:** Pseudo R^2^ and *p* values of Kolmogorov–Smirnov normality test

Method	OLS	Basic GWR	Local linear	CGWR
Pseudo R^2^	0.743	0.953	0.837	0.937
K–S test *p* value	0.906	0.625	0.870	0.772

## Conclusions

The GWR has become a popular tool for explanatory data analysis and detecting spatial non-stationarity ever since its introduction. The GWR provides useful information for data analysis, especially helpful in deciding important explanatory variables. This technique allows regression coefficients to vary across space and obtains their estimates from a bandwidth of observations according the data attribute. However, the GWR tends to produce ragged surfaces (as shown in Fig. 1), and a fixed bandwidth may not be appropriate since the independent variables are necessary to be homogeneous (e.g., their variations can be quite different). In this study, we proposed a modification to the GWR, namely CGWR, which allows the group of positively correlated independent variables to have its own bandwidth via an iterative calibration process.

We used computer simulated and empirical data to compare the proposed method with the GWR and its local linear modification by Wang et al. [31]. Based on the simulation results, we found that the CGWR outperforms other two methods, with respect to the bias and variance, when the regression coefficients are positively correlated. The advantage is especially noticeable in the case of non-linear surfaces. In particular, the clusters have little influences on the estimation of CGWR. The results of empirical studies also support the CGWR and it generally has larger R-square and has fewer extreme outliers (e.g., the absolute value of standardized residual larger than 2 or 3) than the GWR and the local linear method.

However, the proposed method has its limitations. First, probably the most critical limitation, the current setting of CGWR only works for the case if there are independent variables with positive correlation. Although not shown here, we found that the CGWR does not work well in the case of independent variables with negative correlation. It is like the antithetic variate in variance reduction of Monte Carlo Integration. Antithetic variate is one of the popular variance reduction methods but it only works when two variables are negatively correlated [25]. Thus, we suggest first calculating the correlation coefficients between independent variables. Then, form a group of variables which are pairwise positively correlated and apply the CGWR only to this group of variables. Another possibility is that the independent variables often can be separated into two groups and variables within/between groups are positively/negatively correlated, as seen in Taiwan 2000 Census data. We can apply the two-stage CGWR to two groups of variables.

Second, the CGWR is a computer-intensive method and its computing time increases rapidly as the number of variables increases, although the convergence of coefficients can be speed up by using the moving average method. Third, the CGWR is not guaranteed to work if there are many variables, and so far it is effective for the case up to four variables. A possible modification to the case with more variables would be to separate the variables into two groups and use double iteration. Then, the CGWR can be applied to each group of variables forming the inner loop and the process re-iterated between the two groups forming the outer loop until both groups of variables converge. To demonstrate the feasibility of this idea, we also conducted an experiment with six variables, separating them into two groups of three variables each. We found that the estimation did converge and produce satisfactory estimates.

In addition to the fixed bandwidth, it seems that there is still room for improvement about the GWR. In particular, when the S/N ratio is small, the estimated coefficient surfaces would be non-linear (i.e., ragged surfaces), even when the true surfaces are linear. In addition, the variance reduction of the CGWR over the GWR is more obvious than that for bias reduction. This indicates that the GWR estimates have large variance when the S/N ratio is small. In other words, if the variances of GWR estimates are reduced, the bias can also be further reduced, producing more stable estimates.

## Additional file

**Additional file 1.** The outline proof of the convergence for CGWR with fixed bandwidths.
