# Supplementary material for: A modification to geographically weighted regression
Source: Int J Health Geogr. 2017 Mar 31;16:11. doi: 10.1186/s12942-017-0085-9 (PMC5439144; doi:10.1186/s12942-017-0085-9)
Supplement: Supplementary file 1 — Additional file 1. The outline proof of the convergence for CGWR with fixed bandwidths. [file 12942_2017_85_MOESM1_ESM.docx]

Appendix A. The outline proof of the convergence for CGWR with fixed bandwidths

Proposition: *Given that the bandwidths are fixed, let us assume that there is only one independent variable, i.e. there are two coefficients, one intercept, and an explanatory variable. In addition, the bandwidths are fixed during the iteration process. If the largest absolute eigenvalue of the product of two smoothing matrices* ($S_{1} \times S_{2}$) *is less than 1, then the CGWR estimation would converge.*

Outline Proof:

Consider a non-stationary model with only the intercept and a single explanatory variable (i.e.,$Y_{i}=f_{i1}+f_{i2}+\varepsilon_{i}$), where$\varepsilon_{i}$is the random error and$Y_{i}$is outcome of an observation.

To show the convergence of the CGWR algorithm, we first let the initial values be$\hat{f}_{1}(0)=\hat{f}_{2}(0)=0$. Then, the estimation ofcan be written as

$\begin{matrix} \hat{f}_{1}(k)=S_{1}Y-S_{1}S_{2}Y+S_{1}S_{2}S_{1}Y-S_{1}S_{2}S_{1}S_{2}Y+...+R_{1} \\ \hat{f}_{2}(k)=S_{2}Y-S_{2}S_{1}Y+S_{2}S_{1}S_{2}Y-S_{2}S_{1}S_{2}S_{1}Y+...+R_{2} \end{matrix}$ (A1)

whereare the remainder terms of the estimators.

Next, we assume that$Y$,$S_{1}$, and $S_{2}$are non-negative matrix. Then the non-negative properties are straightforward if the explanatory variables are also positive. Note that, the equations of (A1) are alternating series and therefore Leibniz convergence test can be applied. To prove the convergence of the CGWR algorithm, we need to show that both$R_{1}$and$R_{2}$will converge to zero. Since the derivation$R_{2}$is similar, we will only show the derivation for the case of$R_{1}$. The case of$R_{1}$can be separated into two parts.

Let us assume that $(S_{1}S_{2})^{\text{k}^{\text{*}}}=P^{-1}B^{\text{k}^{\text{*}}}P$, where $P$ is a matrix of eigenvectors for $S_{1}S_{2}$at its columns; $B^{\text{k}^{\text{*}}}$is a diagonal matrix having the eigenvalues $S_{1}S_{2}$on the diagonal; and *k** is an arbitrary natural number.

If $\left|\lambda\right|$, the largest eigenvalue amongst the absolute values of $S_{1}S_{2}$ is less than 1, then the remainder$R_{1}$converges to 0 as$k\to\infty.$

The non-negative assumption is set up for simplicity of the proof. This assumption can be archive easily through the variables mean shift. Although no simple proof, however, we find that the series seems to converge for the cases where some variables can be negative. The smoothing matrices in the proof are similar to those used in the GAM. According to Hastie and Tishibirani [16, p.121], the smoother matrices should satisfy the bounded condition. This assumption is also true in practice, and it is always the case in our simulation and empirical studies.

Appendix B. Detailed Results for Bandwidths (Single-type Surfaces)

|  |  | **Linear surface** | | | **Quadratic surface** | | |
| --- | --- | --- | --- | --- | --- | --- | --- |
| **S/N Ratio** | $\beta_{0}$ $\beta_{1}$ | **1** | **3** | **5** | **1** | **3** | **5** |
|  | **1** | 2.35 | 1.57 | 1.35 | 4.03 | 1.13 | 0.98 |
| GWR | **3** | 1.75 | 1.46 | 1.26 | 1.26 | 1.03 | 0.94 |
|  | **5** | 1.56 | 1.31 | 1.20 | 1.09 | 0.98 | 0.92 |
|  | **1** | 9.73 | 9.56 | 10.26 | 2.63 | 1.58 | 1.22 |
| Local  linear | **3** | 10.56 | 10.8 | 10.06 | 1.71 | 1.33 | 1.13 |
|  | **5** | 10.01 | 9.82 | 10.34 | 1.50 | 1.21 | 1.06 |
|  | **1** | 4.16; 7.24 | 1.60; 4.82 | 1.23; 5.19 | 6.58; 7.77 | 0.87; 8.27 | 0.66; 6.28 |
| CGWR | **3** | 4.23; 2.70 | 1.65; 2.31 | 1.18; 2.28 | 6.02; 3.69 | 0.82; 3.82 | 0.61; 2.10 |
|  | **5** | 3.50; 1.70 | 1.60; 1.65 | 1.25; 1.47 | 4.77; 1.44 | 0.89; 1.35 | 0.61; 0.88 |
|  |  | **Ridge surface** | | | **Hillside surface** | | |
|  | **1** | 4.2 | 1.01 | 0.83 | 2.25 | 1.67 | 1.41 |
| GWR | **3** | 1.16 | 0.89 | 0.77 | 1.79 | 1.47 | 1.31 |
|  | **5** | 1.01 | 0.80 | 0.73 | 1.63 | 1.43 | 1.24 |
|  | **1** | 2.61 | 1.33 | 1.03 | 9.78 | 9.78 | 8.2 |
| Local  linear | **3** | 1.68 | 1.12 | 0.95 | 9.75 | 9.01 | 7.4 |
|  | **5** | 1.28 | 1.01 | 0.91 | 8.67 | 8.93 | 6.04 |
|  | **1** | 4.79; 8.79 | 0.85; 8.69 | 0.65; 5.45 | 4.72; 7.45 | 1.63; 5.83 | 1.25; 5.34 |
| CGWR | **3** | 5.24; 4.07 | 0.83; 2.15 | 0.66; 1.12 | 4.98; 3.11 | 1.57; 2.80 | 1.24; 2.70 |
|  | **5** | 3.64; 1.16 | 0.80; 0.99 | 0.65; 0.79 | 5.09; 1.67 | 1.68; 1.64 | 1.25; 1.66 |

Note: CGWR has two bandwidths for two coefficients,$\beta_{0}$and$\beta_{1}$. The values (1, 3, 5) indicate the Signal/Noise Ratio.
